# Dynamics of microbial-induced oil degradation at the microscale

**DOI:** 10.1128/spectrum.01176-24

**Published:** 2024-10-22

**Authors:** Hong Zhang, Wenchao Zhang, Yiwu Zong, Dongyang Kong, Luyan Ma, Xiao-Lei Wu, Kun Zhao

**Affiliations:** 1 Frontiers Science Center for Synthetic Biology and Key Laboratory of Systems Bioengineering (Ministry of Education), School of Chemical Engineering and Technology, Tianjin, China; 2 Petrochemical Research Institute of Petrochina Co., Ltd., Beijing, China; 3 School of Chemistry and Life Science, Suzhou University of Science and Technology, Suzhou, China; 4 State Key Laboratory of Microbial Resources, Institute of Microbiology, Chinese Academy of Sciences, Beijing, China; 5 College of Engineering, Peking University, Beijing, China; 6 Institute of Fundamental and Frontier Sciences, University of Electronic Science and Technology of China, Chengdu, Sichuan, China; 7 Sichuan Provincial People’s Hospital, University of Electronic Science and Technology of China, Chengdu, Sichuan, China; Ruhr-Universitat Bochum, Bochum, Germany

**Keywords:** *Pseudomonas aeruginosa*, oil-water interface, bioremediation, *Dietzia *sp., biofilm

## Abstract

Microbial-induced oil degradation (MIOD) has a wide range of applications, such as microbial enhanced oil recovery and bioremediation of oil pollution. However, our understanding of MIOD is still far from complete. Particularly, how is the dynamics of degradation process at the microscale level with a single-cell resolution remains to be disclosed. In this work, using hexadecane droplets in water as a model system, we have studied the dynamics of hexadecane degradation by different strains, including *Pseudomonas aeruginosa* PAO1, IMP68, O-2-2, and *Dietzia* sp. DQ12-45-1b, at the microscale. Based on visualization of MIOD, the dynamics of MIOD can be characterized by a three-stage process, including adhesion, adaptation, and degradation stages. Although different strains showed similar three-stage dynamics of MIOD, the effective degradation rate varied and followed an order of PAO1 > O-2-2 > IMP68 > DQ12-45-1b under aerobic conditions. Different oxygen conditions were also tested, and the dynamics of MIOD was slowed down under anaerobic conditions in comparison to under aerobic conditions. Further investigations at the degradation stage revealed that biofilms formed at the oil-water interface enhanced oil degradation, but a prerequisite for such enhanced degradation is proper stimulation of biofilm cells in the course of biofilm formation. The findings in this work provided a detailed picture on the dynamics of MIOD at the microscale and would be beneficial for better applications of MIOD.

**IMPORTANCE:**

Microbial-induced oil degradation is environmental friendly and economic and has become a promising technique in the fields of enhanced oil recovery and remediation of crude oil-polluted environments. For better applications of microbial-induced oil degradation, understanding the degradation dynamics particularly at the microscale is crucial. In this study, we investigated the degradation dynamics of hexadecane oil droplets incubated with different strains, including *Pseudomonas aeruginosa* PAO1, O-2-2, IMP68, and *Dietzia* sp. DQ12-45-1b at the microscale by employing microdroplet-based methods and bacterial tracking techniques. The findings in this study provided a detailed picture on the dynamics of microbial-induced oil degradation at the microscale, which will deepen our understandings on the biodegradation mechanisms of alkanes and shed insights for developing more effective biodegradation techniques.

## INTRODUCTION

The continuous leakage of crude oil poses a significant threat to ecosystems and human being (1). Thus, how to effectively address the environmental problems caused by petroleum hydrocarbons pollution is currently a serious challenge (2). Microbial-induced oil degradation (MIOD) is environmental friendly and economic and has become a promising technique in the fields of enhanced oil recovery (3) and remediation of crude oil-polluted environments (4).

Quite a few strains of bacteria that use crude oil as their sole carbon sources have been isolated (2) and further utilized for oil degradation, such as *Streptococcus* (4), *Acinetobacter* (5), *Alcaligenes* (6), *Dietzia* (7) and *Pseudomonas* (8). Among them, *Pseudomonas aeruginosa* (*P. aeruginosa*) and *Dietzia* spp. exhibit high performance in biodegradation due to their excellent capabilities in degrading long-chain alkanes and producing surfactants (9). Many studies from different perspectives of MIOD have been reported, including screening of petroleum-degrading bacteria (10), metabolic pathway research (11), immobilization of microorganisms (12), biological stimulation (13, 14), and factors affecting the degradation of petroleum hydrocarbons (15–22). However, only a few studies on MIOD have focused on the microscopic scale, such as observing the morphology of oil droplets under microscopic conditions to separate strains that can biodegrade petroleum hydrocarbons (23), or measuring the hydrophobicity and biofilm formation of these bacteria at the liquid-liquid interface through interfacial rheology and pendant drop tensiometry (24). A recent study at the microscale revealed that after the biodegradation of alkanes, different biofilm structures were observed for different strains used (25). Despite these advances, given the relatively few research at the microscale, our understanding of MIOD is still far from complete. Particularly, how is the dynamics of degradation process at the microscale level with a single-cell resolution remains to be disclosed.

To study MIOD at the microscale, common methods used for measuring petroleum degradation behavior are not enough, such as gas (liquid) chromatography (26, 27), cell relative hydrophobicity test [bacterial adherence to hydrocarbons (BATH) test] (28, 29), interfacial tension test (30), and fluorescence measurement of petroleum hydrocarbon staining components (31). Bacterial tracking techniques together with droplet-based microfluidics have been shown to be able to monitor the evolution of bacterial cells at the oil-water interface during the degradation of oil droplets at the single-cell level (32), thus providing a suitable route for studying the dynamics of MIOD at the microscale.

In this study, we used hexadecane droplets in water as a model system to understand the degradation process of different bacteria, including *P. aeruginosa* (PAO1, O-2-2, IMP68) and *Dietzia* sp. DQ12-45-1b at the hexadecane-water interface. Among the four strains, IMP68, O-2-2, and DQ 12–45-1b are originally isolated from oil-field environments, while PAO1 is a laboratory model strain and acts as a control strain for IMP68 and O-2-2. DQ12-45-1b has been reported to be able to degrade hexadecane (9, 33), while there are also studies suggesting that *P. aeruginosa* can utilize C12-C38 as the sole carbon source (34–37). So, understanding their MIOD is not only scientifically interest but also of importance in applications. In addition, different from *P. aeruginosa* that is gram negative, DQ12-45-1b is gram positive. Thus, including DQ12-45-1b would enrich our understandings of MIOD.

To study MIOD, micron-sized hexadecane droplets were first prepared and stabilized on glass surfaces. Then, real-time *in situ* monitoring of the hexadecane degradation process by selected bacteria at the hexadecane-water interface was conducted using bacterial tracking techniques. Through directly observing the degradation process of hexadecane droplets by different bacteria under aerobic and anaerobic conditions, the dynamics of MIOD were visualized and characterized. By comparing degradation behavior of different strains under different conditions, factors that affect the dynamics of MIOD were also discussed. The findings in this study provided a detailed picture on the dynamics of MIOD at the microscale, which will deepen our understandings on the biodegradation mechanisms of alkanes and shed insight into developing more effective biodegradation techniques.

## RESULTS

### Characterization of MIOD at the microscale revealed three stages of MIOD dynamics

**Fig 1:**
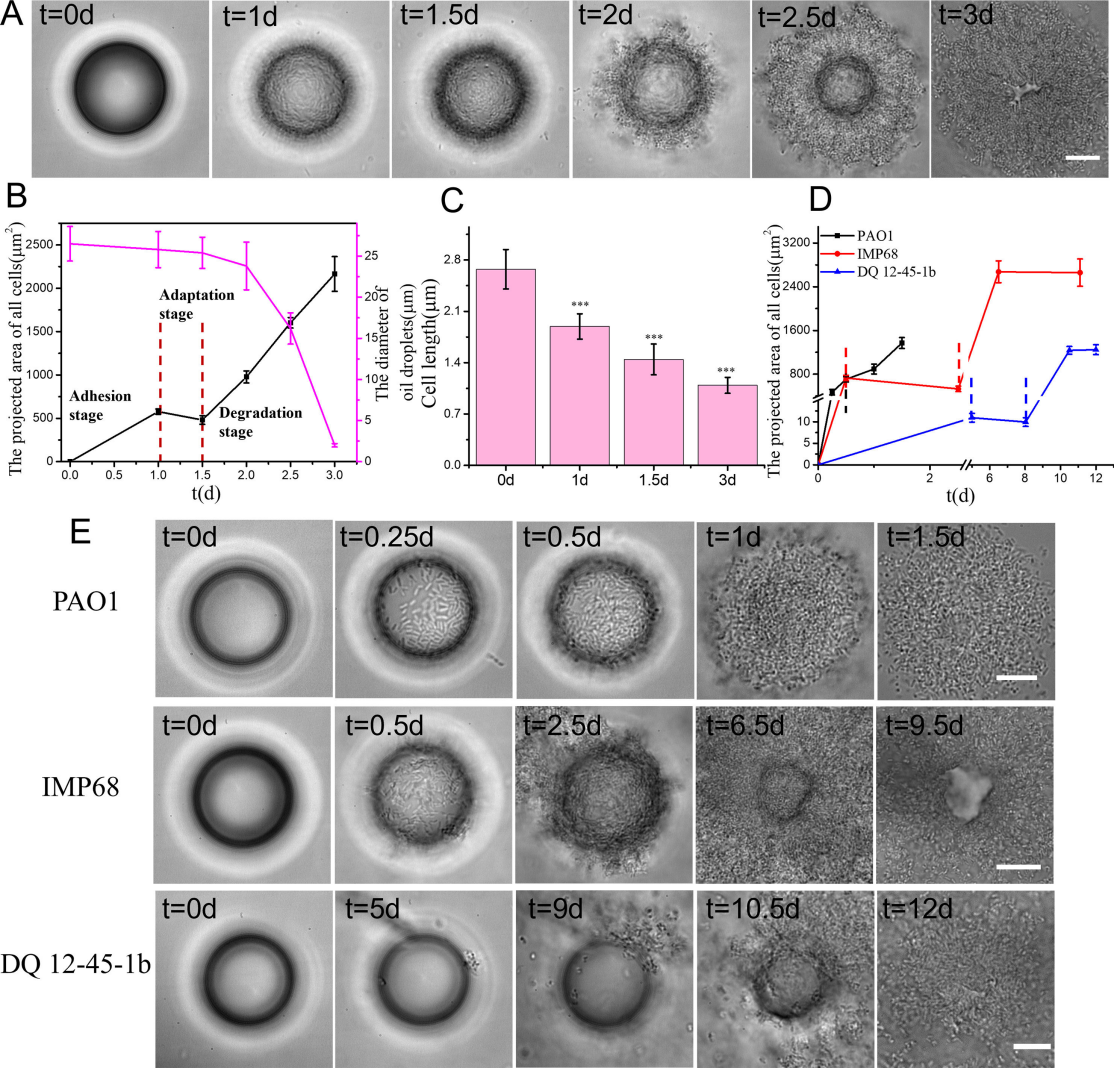
Characterization of MIOD at the microscale for different strains. (**A**) Snapshots taken at different time points during the degradation process of a hexadecane droplet by *P. aeruginosa* O-2-2. (**B**) The projected area of all cells that were associated with the oil-water interface and the diameter of the oil droplet measured at different time points for *P. aeruginosa* O-2-2. (**C**) The cell length measured at different time points for *P. aeruginosa* O-2-2. (**D**) Changes in the projected area of all cells associated with the oil-water interface over time, for PAO1, IMP68, and DQ12-45-1b. Different stages of bacterial degradation of alkanes were indicated by dashed lines. (**E**) Snapshots taken at different time points during the degradation process of a hexadecane droplet for *P. aeruginosa* PAO1 (top row), IMP68 (middle row), and *Dietzia* sp. DQ12-45-1b (bottom row). Statistical significances were measured using a one-way analysis of variance set for multiple comparisons with a Dunnett’s post test. *** *P* is very highly significant at *P* < 0.001. The analysis of statistical significance was performed between 0 days and other time points. Error bars represent the standard deviations of the mean of three oil droplets. Scale bar, 10 µm.

Figure 1A shows snapshots of an oil droplet captured at different time points when it was incubated with *P. aeruginosa* O-2-2, which demonstrated a whole microbial-induced degradation process of an oil droplet that started from initial bacterial inoculation to the final state when the entire oil droplet was depleted. Quantitatively, we can characterize the entire process by monitoring the projected area of cells that were associated with the oil-water interface as well as the cell length as a function of time. The results are shown in Fig. 1B and C. Based on these results, the dynamics of MIOD can be divided into three stages: adhesion, adaptation, and degradation stages. During the adhesion stage, which lasted for about 1 day after bacterial inoculation (defined as *t* = 0), most of cells in the sample solution adhered to the oil-water interface. Thus, the droplet surface gradually changed from a smooth looking to a golf-ball-like looking. At the end of 1 day, the droplet size was slightly reduced from 26.5 ± 2.1 µm at *t* = 0 days to 25.8 ± 2.2 µm at *t* = 1 day. Then, during the next 0.5 days, cells stably attached to the oil-water interface, but the total area of attached cells slightly decreased, from 577 ± 31 to 481 ± 50 µm^2^ (Fig. 1B), indicating that during this time period, cell growth was negatively affected. This is also in agreement with the cell length measurement, which was reduced significantly from 2.7 ± 0.3 µm at *t* = 0 days to 1.4 ± 0.2 µm at *t* = 1.5 days. We speculate this cell shrinkage may be due to inadequate nutrition in the environment. To test this hypothesis, we added yeast extract (5 g/L) as additional nutrients to the sample pool that originally containing hexadecane as the sole carbon source. Cells at the oil-water interface could rapidly grow and form biofilms. Before biofilm formation (i.e., <4 h), the cell length increased from 2.38 ± 0.53 µm (*t* = 0 h) to 3.75 ± 0.81 µm (*t* = 3 h), as shown in Fig. S1A. These observations support our hypothesis that cell size reduction is likely due to inadequate nutrition. We named this growth-halting period from *t* = 1 day to t = 1.5 days as the adaptation stage, given the fact that cells would resume their growth in the following degradation stage. During the degradation stage, cells were observed to be able to grow and multiply presumably through oil degradation to obtain the necessary carbon nutrients. As bacterial cells continued to multiply, cell population at the oil-water interface became denser and later even extended out to a much bigger region than the original droplet size. Apparently, a biofilm developed during the degradation stage. At the end of degradation, the cell length was 1.1 ± 0.1 µm (Fig. 1C), which is smaller than that at *t* = 1.5 days. Similar cell size reduction was also observed in a control experiment where the biofilm formation of O-2-2 on a glass surface was monitored using a flow-cell device supplemented with FAB medium and 0.6 mM glutamate. The results (Fig. S1B) showed that the size of O-2-2 cells reduced from 2.80 ± 0.24 µm at *t* = 3 h before biofilm developed (*t* = 6 h) to 1.60 ± 0.25 µm (*t* = 9 h) after biofilm developed. This is likely caused by the stresses due to, for example, pressure from crowded cell population (38) or depletion of nutrient sources. Accompanied with cell multiplication, the droplet became smaller with time and was nearly depleted at the end of degradation.

### Different strains showed similar three-stage dynamics of MIOD but with different degradation efficiencies

To understand the dependence of the dynamics of MIOD on different strains, besides *P. aeruginosa* O-2-2, we also measured the degradation behavior of *P. aeruginosa* PAO1, IMP68, and *Dietzia* sp. DQ12-45-1b on hexadecane droplets under aerobic conditions. The results show that the similar three stages of MIOD were observed in all tested strains (Fig. 1D and E). However, the duration time of each stage varied significantly among different strains. For example, IMP68 has a duration of 1.23 ± 0.01 days for the adhesion stage, 1.28 ± 0.01 days for the adaptation stage, and 8.59 ± 0.04 days for the degradation stage, while the corresponding values of DQ12-45-1b are 5.00 ± 0.04, 3.03 ± 0.02, and 4.05 ± 0.02 days, respectively. For PAO1 cells, the whole degradation process from cell attachment to the depletion of oil droplet only took about 1.5 days under our conditions. With such a quick degradation process, the adaptation stage of PAO1 cells is not easy to be detected.

**Fig 2:**
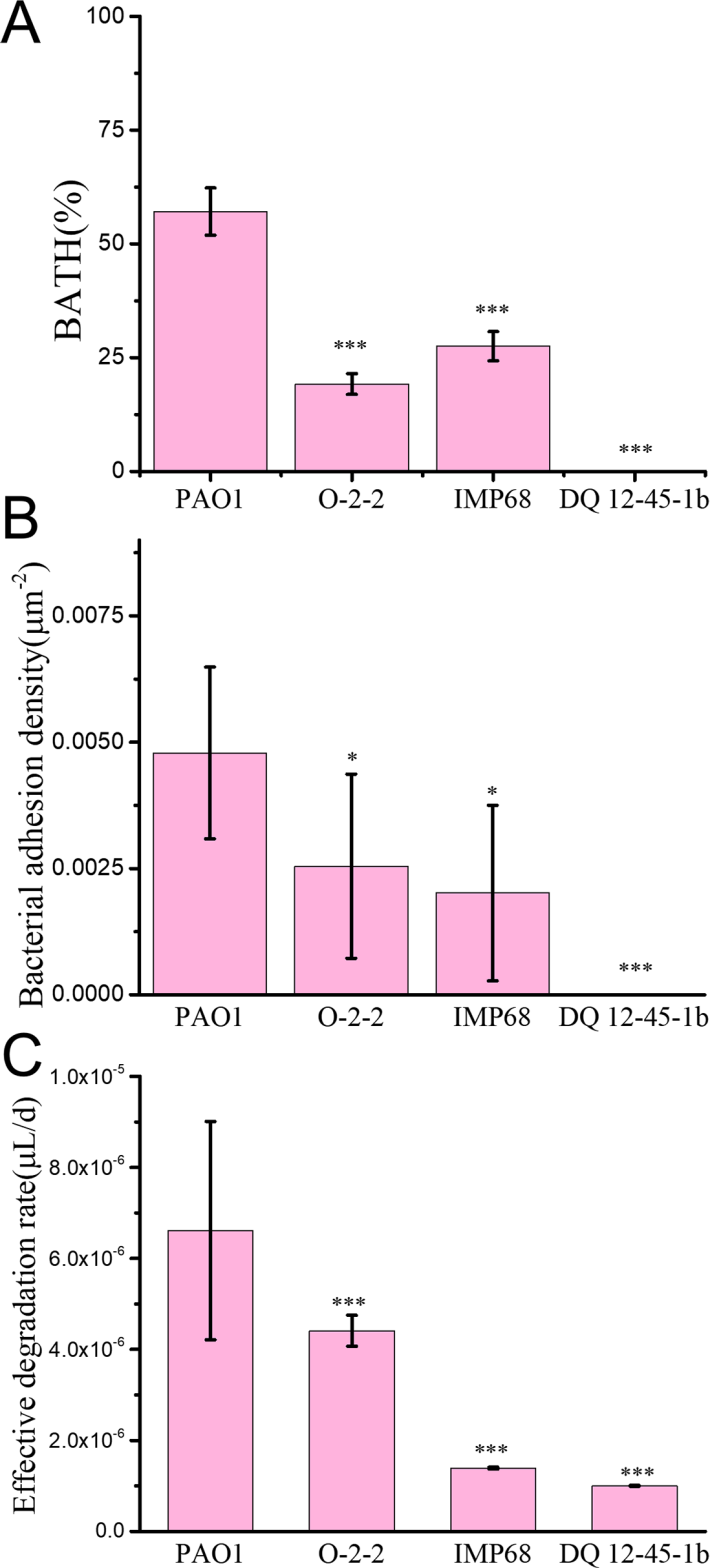
(**A**) BATH measurement and (**B**) bacterial adhesion densities on hexadecane oil droplets for different strains; (**C**) effective degradation rate. The analysis of statistical significance was performed between PAO1 and other strains using a one-way ANOVA set for multiple comparisons with a Dunnett’s post test. *P < 0.05 and ***P < 0.001. Error bars represent the standard deviations of the mean of three oil droplets.

From Fig. 1E, we also noticed that DQ12-45-1b cells seemed to have difficulties to be trapped at the oil-water interface, and even at t = 5 days, only a small portion of bacteria cells (with a projection area of only 10.93 ± 1.01 µm^2^) adhered to the oil-water interface (Fig. 1D). To understand the differences in cell attachment to the oil-water interface, we measured the hydrophobicity of cell surfaces for each strain using two methods, bacterial adherence to hydrocarbons method performed at the macroscale and bacterial adhesion density at the oil-water interface measured 1 hour after inoculation through bacterial tracking techniques. The results are shown in Fig. 2A and B. Both measurements showed that PAO1 had the highest adhesion value to hexadecane, while DQ12-45-1b had the lowest one. These results are consistent with the degradation performance summarized in Table 1. We note that IMP68 and O-2-2 showed opposite trends in the adhesion behavior measured by the two testing methods. BATH showed that IMP68 had a higher affinity for hexadecane than O-2-2, while the bacterial adhesion density measurements were just opposite. The reason for the difference is likely due to the fact that O-2-2 cells were observed to be able to swim, while IMP68 cells were not under our conditions (data not shown). For the BATH test, bacterial adhesion was a passive process, in which the fluid flow is mainly responsible for taking cells to the oil-water interfaces, and bacterial motility has a minor role. However, for the bacteria adhesion density measurement, there was no fluid flow generated by external forces, and bacterial motility became important, as motile cells can have more chances to move toward the oil-water interfaces. Thus, the non-swimming phenotype of IMP68 cells resulted in a decrease in their adhesion density compared with O-2-2 cells.

**TABLE 1:** Dynamics of MIOD under aerobic and anaerobic conditions for different strains^*a*^

Strain	Adhesion time (days)	Adaptation time (days)	Degradation time (days)	Effective degradation rate (× 10^−6^ µL/d)
Aerobic				
PAO1	0.27 ± 0.01	0.24 ± 0.01	1.04 ± 0.02	6.61 ± 0.24
O-2–2	1.01 ± 0.03	0.51 ± 0.01	1.53 ± 0.01	4.41 ± 0.34
IMP68	1.23 ± 0.01	1.28 ± 0.01	8.59 ± 0.04	1.39 ± 0.02
DQ12-45-1b	5.00 ± 0.04	3.03 ± 0.02	4.05 ± 0.02	1.00 ± 0.01
Anaerobic				
PAO1				^*b*^
O-2–2	1.02 ± 0.02	11.19 ± 0.02	3.33 ± 0.01	0.92 ± 0.03
IMP68	1.25 ± 0.04	12.28 ± 0.02	2.68 ± 0.01	0.82 ± 0.02
DQ12-45-1b				*^b^*

^*a*^ The presented values were averaged from three oil droplets.

^*b*^ Did not observe significant reduction of oil droplets even after 60 days of incubation.

To characterize the efficiency of bacterial degradation of oil droplets, we can calculate the effective degradation rate by dividing the total volume of an oil droplet by the total duration time of the three stages. Thus, this effective degradation rate also includes the effect of cell behavior at the adhesion and adaptation stages. Although from the perspective of metabolization activities, the degradation rate calculated by dividing the volume of oil droplet at the beginning of degradation stage by the degradation time would be more appropriate, the effective degradation rate would be more practical since macroscale studies typically do not differentiate these three stages. The results are shown in Fig. 2C (also summarized in Table 1). We can see that among the tested strains, the effective degradation rate followed an order of PAO1 > O-2-2 > IMP68 > DQ12-45-1b.

### The dynamics of MIOD was slowed down under anaerobic conditions, and the extent to be slowed varied among tested strains

As strains O-2-2 and IMP68 were originally obtained from oil-reservoir environment where cells often stay under low or without oxygen conditions, we then performed the oil degradation tests under anaerobic conditions to reveal the effect of oxygen on the dynamics of MIOD. The results are shown in Table 1. Under the tested anaerobic conditions, DQ12-45-1b and PAO1 cells did not show apparent growth, and no degradation of oil droplets was observed even after 60 days of incubation.

For O-2-2 and IMP68, they all showed a much longer adaptation time under anaerobic conditions than under aerobic conditions, which led to a much smaller effective degradation rate accordingly. However, compared with O-2-2, whose oil degradation performance was better under aerobic conditions than under anaerobic conditions at each stage of MIOD, IMP68 displayed a shorter degradation time under anaerobic conditions (2.68 ± 0.01 days) than under aerobic conditions (8.59 ± 0.04 days). But because IMP68 had a much longer adaptation time of 12.28 ± 0.02 days under anaerobic conditions compared with 1.28 ± 0.01 days under aerobic conditions, the overall effective degradation rate was lower under anaerobic conditions (0.82 ± 0.02 × 10^−6^ µL/d) than under aerobic conditions (1.39 ± 0.02 × 10^−6^ µL/d). These results indicate that from the perspective of the degradation capability itself, IMP68 performed better without oxygen than with oxygen. But to reveal its specific mechanism, further investigations are needed.

### Biofilms formed at the oil-water interface during the degradation stage of MIOD enhanced oil degradation

Since biofilms were formed during the degradation stage, during which the entire oil droplet was being biodegraded completely, we further characterized biofilm properties to reveal their effects on the oil degradation. Table 2 listed the measured thickness and biomass surface coverage for O-2-2 and IMP68 under both aerobic and anaerobic conditions. Under aerobic conditions, a biofilm of O-2-2 had a thickness of 23.3 ± 1.3 µm and a biomass surface coverage of 76.9% ± 7.6%, while IMP68 formed a biofilm with a thickness of 15.6 ± 2.6 µm and a biomass surface coverage of 41.6% ± 5.7%. Under anaerobic conditions, the corresponding values of biofilm thickness and biomass surface coverage were 18.9 ± 1.2 µm and 55.1% ± 4.4% for O-2-2, and 23.8 ± 1.6 µm and 100.0% ± 0.0% for IMP68. So biofilms of O-2-2 formed under aerobic conditions are thicker and have larger surface coverage than those under anaerobic conditions, while IMP68 biofilms were just the opposite. As shown in Table 1, when compared between aerobic and anaerobic conditions, the degradation time of O-2-2 is shorter under aerobic conditions but that of IMP68 is shorter under anaerobic conditions. Taken together, these results indicate a negative correlation of biofilm thickness and biomass surface coverage vs the degradation time. The thicker and more surface covered a biofilm, the shorter the degradation time (i.e., oil degradation is faster).

**TABLE 2:** Biofilm thickness and biomass surface coverage measured at the end of the degradation of hexadecane droplets by O-2-2 and IMP68 under aerobic and anaerobic conditions

Culture conditions	Strain	Biofilm thickness (µm)	Biomass surface coverage (%)
Aerobic	O-2-2	23.3 ± 1.3	76.9 ± 7.6
	IMP68	15.6 ± 2.6	41.6 ± 5.7
Anaerobic	O-2-2	18.9 ± 1.2	55.1 ± 4.4
	IMP68	23.8 ± 1.6	100.0 ± 0.0

**Fig 3:**
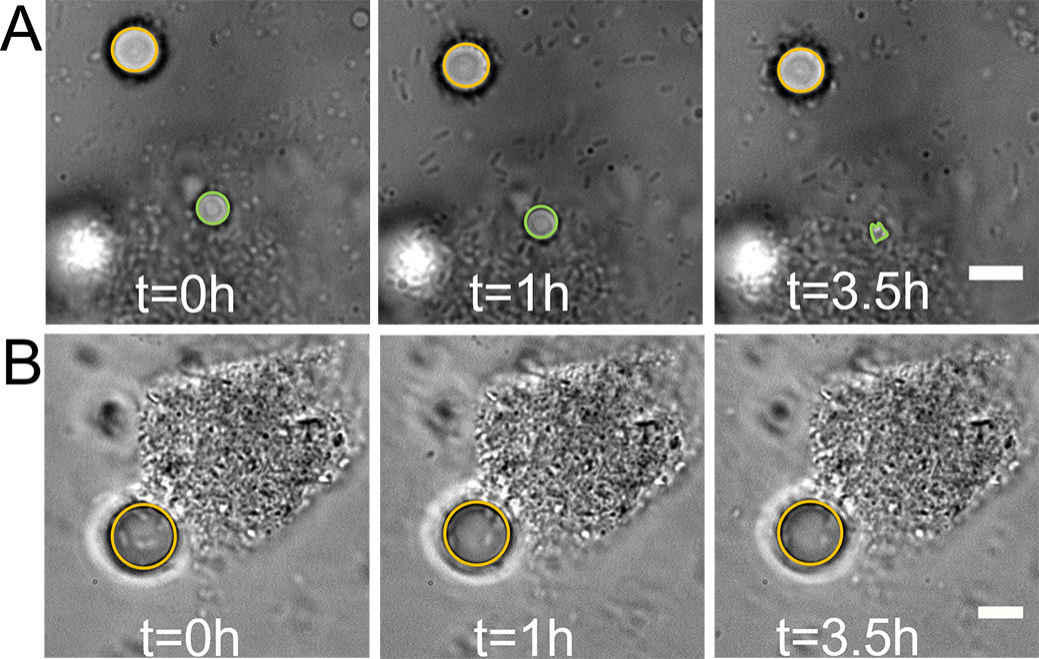
Oil degradation tests of stimulated planktonic cells and cell aggregates/biofilms. (**A**) Degradation of hexadecane at three time points by planktonic cells and cell aggregates/biofilms. The oil droplet indicated by the green circle was degraded by cell aggregates/biofilms, while the one indicated by the orange circle was degraded by planktonic cells. (**B**) Degradation of hexadecane at three time points by cell aggregates/biofilms formed with glucose as the sole carbon source. The oil droplet was indicated by an orange circle. Scale bars, 5 µm.

During biofilm development at the oil-water interface, cells in the biofilm were also stimulated as they were being incubated in an environment with the oil as the only available carbon source. To delineate the effect of biofilm from that of cell training on alkane droplet degradation, we performed oil degradation tests using either stimulated single cells or stimulated cell clusters. To do so, taking *P. aeruginosa* O-2-2 as an example, cells from the bacterial culture were observed to exist in two types of forms, planktonic cells (dispersed individual ones) and cell aggregates/biofilms. Since they were from the same bacterial culture, cells in these two forms would have same stimulating history. We found that for fresh oil droplets surrounded by cell aggregates/biofilms, the degradation process was rapid and did not display all three stages shown in Fig. 1, while for fresh oil droplets surrounded by planktonic cells, the degradation followed the normal three stages and thus was much slower compared with the case of cell aggregates/biofilms. Examples were shown in Fig. 3A. These results indicate that compared with planktonic states, biofilms can indeed better facilitate the degradation of hexadecane. The observation that stimulated cell aggregates/biofilms did not show typical adaptation stage is mainly due to the fact that these cells have been already adapted to the environment with hexadecane as the only carbon source. We suspected that this would not be the case if the cell aggregates/biofilms were pre-entrained in different environments with different available carbon sources. To test this hypothesis, we cultured O-2-2 in a medium with glucose as the sole carbon source and then followed the same procedure as above to inject bacterial culture into a fresh sample pool of hexadecane to observe the degradation of hexadecane droplets under these conditions. The results showed that at *t* = 3.5 h, there was no sign to tell that the oil droplet was degraded (Fig. 3B). We also tested a longer incubation time of 12 h, and still no discernable degradation of hexadecane could be observed. In summary, these results reveal that the formation of cell aggregates/biofilms could contribute to the degradation of hexadecane by O-2-2, but it also depends on the formation environment under which such cell aggregates/biofilms developed.

## DISCUSSION

Understanding the dynamics of microbial-induced oil degradation at the microscale can better facilitate the applications of MIOD techniques in the remediation of crude oil-polluted environments. However, to perform such microscale studies, appropriate techniques that can monitor the cell behavior at the oil-water interface for a long-enough time period (e.g., a time period to cover the depletion of oil droplets in this study) and also in a real-time and *in situ* fashion are needed. These technical challenges are also part of reasons that relatively few studies have been reported so far in the literature on the MIOD dynamics at the microscopic level. In this study, by employing microdroplet-based techniques, we prepared tens-micron-sized alkane oil droplets that attached to a glass surface. The size of these oil droplets is suitable for microscopic observations at high magnifications, where while a whole droplet is visible within the field of view, single bacterial cells at the droplet surface can still be clearly discerned. Since these droplets were attached to the glass surface, they were relatively fixed (i.e., did not diffuse or drift away to be out of the field of view), which enabled us to monitor the morphological evolution of the same droplet during MIOD for a long-enough time. Using bacterial tracking techniques, bacterial behavior at the oil-water interface can be analyzed. Similar methods have also been reported in a recent study by Prasad et al. (25), where the authors prepared droplets of relatively suitable size based on microfluidic methods and observed the degradation process of hexadecane oil droplets by *Alcanivorax borkumensis*. They showed that its sufficient degradation of alkanes was achieved by expanding the contact area with the oil droplets to form dendritic biofilms.

In this study, different strains, including *P. aeruginosa* O-2-2, PAO1, IMP68, and *Dietzia* sp. DQ12-45-1b, were tested. Under aerobic conditions, their degradation process of hexadecane oil droplets follows similar trend and can be divided into three stages: the adhesion stage, the adaptation stage, and the degradation stage (Fig. 1). However, their effective degradation rate varied greatly, which followed the order of PAO1 > O-2-2 > IMP68 > DQ12-45-1b. The difference in the effective degradation rate is related to the initial adhesion efficiency, the adaptation time, and the degradation time. Although the physically decomposing of oil droplets was mainly performed during the degradation stage, the adhesion and adaptation stages are also important for the degradation of hexadecane droplets observed in this study, as the performance of cells during these two stages will lay a foundation for the bio-decomposition of hexadecane during the following degradation stage.

During the adhesion stage, cells took time to be trapped at the oil-water interface through swimming and/or diffusion, so factors that can affect the adhesion of cells at the oil-water interface will also have an influence on the final effective degradation rate. For instance, it has been shown that bacteria with stronger surface hydrophobicity would have better adhesion ability at the oil-water interface (39, 40). In our study, DQ12-45-1b cells had the most hydrophilic cell surfaces among the tested strains, which make them difficult to be trapped at the oil-water interface and thus displayed the longest adhesion time. This contributes largely to the finally obtained much less effective degradation rate. By contrast, PAO1 cells were just opposite. They were more hydrophobic and had the shortest adhesion time, which greatly sped up the whole degradation process. In addition to the hydrophobicity of cell surfaces, cell motility can also contribute to the cell adhesion at the oil-water interface, as it is reasonable to imagine that motile cells would have more chances to move close to the interface and thus increase the probability to be trapped, while non-motile cells may only depend on thermal diffusion or fluid flow. In fact, studies at the macroscale on MIOD were often performed involving shaking samples at a certain rotational speed, which increases the probability of passive contact of bacteria with degradation products and maintains the oxygen level of the samples. In this case, whether cell being motile or not may not be critical as fluid flows generated by shaking can bring cells close to the oil-water interface. This is not the case for the microscopic study presented in this work. Under our conditions, there is no such fluid flow, and bacterial cells have to rely on their motility and/or diffusion to move around. Such differences in the fluid flow together with cell motility also caused the opposite trend of O-2-2 and IMP68 for their hydrophobicity measurements obtained by BATH and bacterial adhesion density methods (Fig. 2).

After the adhesion stage, cells were trapped at the oil-water interface and would adapt to this environment. During the adaptation stage, bacteria did not show apparent growth, and the oil droplet did not vary much. In fact, the cell length reduced significantly at the end of this stage, indicating that cell growth was severely affected during adaptation. Considering that cells would resume to grow in the following degradation stage, we hypothesized that cells during the adaptation stage would change their gene expressions, for example, to upregulate expressions of alkane degradation-related genes, in order to survive in an environment with the hexadecane as the only available carbon source. This hypothesis is also supported by a previous work showing that within the first hour after *P. aeruginosa* contacted with alkanes, for cells at the hexadecane-water interface, their alkane hydroxylase *alkB2* gene involved in the oxidation and decomposition metabolism of C_12_-C_16_ alkanes was induced to be highly expressed (~11.3-fold) (16).

After the adhesion and adaptation stages, cells were ready to use hexadecane as the only carbon source, so during the degradation stage, cells resume growth and division, which resulted in an increased cell density at the oil-water interface. Accompanied by cell multiplications, the droplet shrank with time and finally was depleted (i.e., disappeared in the field of view). The cell multiplication finally led to biofilm formation, which greatly enhanced the biodegradation of hexadecane. Our results showed that the thickness of biofilms positively correlated with degradation efficiency of oil droplets, which is presumably related to high cell number densities and large amount of extracellular substances contained in the biofilms. A macroscopic study had demonstrated that biofilms could promote alkane degradation, mainly due to the emulsifying function of abundant extracellular polysaccharides that they contained (41). Our observations are consistent with this result. However, in order to take advantage of biofilms in biodegradation of oil droplets, they need to be stimulated properly. We showed that for biofilms developed in a culture medium with glucose as the sole carbon source, they did not degrade hexadecane droplets during the tested time period when these biofilms were transferred to the medium with hexadecane as the only available carbon source (Fig. 3).

The whole MIOD process is largely dependent on the oxygen level. As shown in Table 1, generally cell behavior varies according to oxygen levels. The different performance of biodegradation between aerobic and anaerobic conditions is presumably due to different metabolic pathways at the molecular level. Studies on the metabolic pathways of microbial degradation of petroleum hydrocarbons have been reported (42–44). Under aerobic conditions, alkanes enter the cell and undergo the tricarboxylic acid cycle with oxygen as the final electron acceptor, then ultimately are metabolized into carbon dioxide and water (42). While under anaerobic conditions, bacteria utilize alternative electron acceptors such as sulfate, nitrate, iron, manganese, or carbon dioxide to metabolize alkanes via processes activated by CoA, decarboxylation, and multiple steps of β-oxidation (43, 44). The different metabolic pathways used by strains under different oxygen conditions will affect the growth rate of cells and the formation of biofilms, which can be seen in the varied biofilm thickness and biomass surface coverage illustrated in Table 2.

In conclusion, we have studied the dynamics of MIOD and factors that can affect it at the microscale by employing microdroplet-based methods together with bacterial tracking techniques. The findings in this work not only deepen our understanding on MIOD at the microscale but also shed insight into developing strategies to improve the efficiency of MIOD, such as modifying cell surface hydrophobicity/cell motility to reduce bacterial adhesion time and enhancing degradation-associated gene expressions and biofilm formation to speed up the degradation stage.

## MATERIALS AND METHODS

### Strain culture

The strains used in this study are listed in Table S1 in Supporting Information, including *P. aeruginosa* PAO1, IMP68, O-2-2, and *Dietzia* sp. DQ12-45-1b. For each strain, a monoclonal colony on LB agar plate was inoculated into an LB liquid medium, which was then incubated on a shaking table at 37℃ until the bacterial culture reached logarithmic metaphase. We diluted the bacterial culture to appropriate optical density of the water phase at 600 nm wavelength (OD_600_ = 2.0 in this study) and washed it three times with minimal salt medium (MSM) [0.5 g/L KH_2_PO_4_, 1.26 g/L Na_2_HPO_4_·12H_2_O, 3 g/L (NH_4_)_2_SO_4_, 0.54 g/L MgSO_4_·7H_2_O, 1 mL/L trace element solution (0.15 mg/L MnSO_4_·H_2_O, 0.5 mg/L FeCl_3_·6H_2_O, 0.24 mg/L ZnSO_4_·7H_2_O, 3.66 mg/L CoCl_2_·6H_2_O, 0.3 mg/L FeSO_4_·7H_2_O), pH 7.0 ± 0.2]. A 10 µL of bacterial solution was injected into the sample pool for microscopic observations. The detailed protocols were the same as in our previous work (32).

### Preparation of sample pool

The sample pool was prepared in the same way as in literature (32). To prepare hexadecane droplets, a capillary glass tube (2 × 0.1 × 5 mm) was fixed in the center of a 24 × 60 mm cover glass. Then, 2.5 mL of hexadecane was injected into the tube, and some hexadecane would flow out of the tube. One end of the capillary tube was sealed with a photosensitive adhesive. Next, a poly(methyl methacrylate) ring (inner diameter 18 mm, outer diameter 26 mm) was attached to a cover glass using a photosensitive adhesive around the capillary tube, and then 1 mL MSM medium was filled. Due to mechanical interference caused by medium injection, hexadecane droplets (possibly mainly from the outflow of hexadecane) were generated and adhered to the glass surface. The generated hexadecane droplets have a flat bottom when attached to the glass surface, so the upper hemisphere of the hexadecane droplets was selected for experimental measurement, which also minimized the effect from the glass surface on the oil-water interfacial behavior of bacteria. Under aerobic conditions, the top of the adhesive sample cell was open, while under anaerobic conditions, the adhesive sample cell was covered with a 22 × 22 mm glass plate at the top and was sealed with Vaseline. To avoid the effect of solution evaporation under aerobic conditions, sterile deionized water was injected into the sample cell using a syringe pump at a flow rate of 100 µL/h. To monitor the anaerobic condition in a sealed sample pool, an anaerobic indicator, Azuron, was added to the sample pool to indicate the oxygen level by the color changes of culture medium. After the sample pool had been sealed for 1.5 days, the color of the culture medium changed from blue to pink, implying that the oxygen content of the sealing sample was extremely low. Given that the measured degradation period typically was on the order of tens of days, the effect due to the presence of small amount of oxygen in the first 1.5 days would be minor and would not affect the experimental trends reported in this study.

### Bacterial adherence to hydrocarbons

The relative hydrophobicity of different bacterial strains was measured using bacterial adhesion tests to hydrocarbons following the protocols of Rosenberg (29). In short, bacterial suspension was mixed with hexadecane in a volume ratio of 1:1.2 and vortexed for 2 minutes. The obtained mixture was phase separated into water and oil phases after standing still for 15 minutes, and OD_600_ was measured. To quantify bacterial adhesion to the oil phase, the ratio of optical density before and after mixing was calculated as BATH = 100 × (1 − OD_600_ after mixing/OD_600_ before mixing), which is commonly referred to as one metric for measuring bacterial hydrophobicity.

### Cells stimulated by hexadecane or glucose

When *P. aeruginosa* O-2-2 was stimulated and cultured with hexadecane as the sole carbon source, an MSM medium with a hexadecane concentration of 2% (vol/vol) was used. O-2-2 cells were first cultured with hexadecane as the sole carbon source in a shaking flask for 6 days. Next, we washed the bacterial culture three times using MSM medium, then a 20 µL sediment from bacterial culture medium was pipetted from the flask and injected into a fresh sample pool that contained 1 mL MSM medium with hexadecane, which was then examined under microscope. For replacing hexadecane with glucose as the sole carbon source in MSM, a final glucose concentration of 10 g/L was used.

### Data processing and analysis

Data were collected using an inverted fluorescence microscope equipped with a camera. The captured image size is 66.49 × 66.49 µm. To record the degradation process of oil droplets, the selected oil droplet was scanned with a step size of 0.5 µm in *z*-direction (i.e., vertical direction) at chosen time points. From the recorded images, the diameter and, hence, volume of the oil droplets can be determined. To avoid possible influences of the droplet size on the results (Fig. S2), in this study, only droplets that have a size within the range of 25 ± 2 µm were selected for analysis. The recording would continue until the oil droplets in the field of view were completely degraded or the total experimental time reached a certain value (60 days). For each degradation experiment, we selected three oil droplets for analysis.

When oil droplets were completely degraded, the biofilms originated from the oil-water interface were characterized. The thickness of a biofilm was measured by *z*-scanning with a step size of 0.5 µm. For the biomass surface coverage measurement, we first selected an image window with a size of 25 × 25 µm centered on an oil droplet (i.e., selecting a square area within the range of the oil droplet), which is 5 µm higher than the glass surface. Then, we used Image J to measure the biomass surface coverage as the ratio of the total area of biofilm cells to the area of the selected image window (i.e., 25 × 25 µm). The projected area of bacteria associated with droplets and the length of individual cells were measured by Image J. By analyzing the change in the projected area, the adhesion, adaptation, and degradation stages of MIOD were determined. The effective degradation rate was calculated by dividing the volume of a droplet by the total degradation time (sum of the duration time of three stages), which is different from the degradation rate calculated as the total volume of a droplet divided by the duration time of the degradation stage only.

## SUPPLEMENTAL MATERIAL

Supplemental material spectrum.01176-24-s0001.docx Table S1; Fig. S1 and S2.

ASM does not own the copyrights to Supplemental Material that may be linked to, or accessed through, an article. The authors have granted ASM a non-exclusive, world-wide license to publish the Supplemental Material files. Please contact the corresponding author directly for reuse.
