# Supplementary material for: Dynamics of microbial-induced oil degradation at the microscale
Source: Microbiol Spectr. 2024 Oct 22;12(12):e01176-24. doi: 10.1128/spectrum.01176-24 (PMC11619474; doi:10.1128/spectrum.01176-24)
Supplement: Supplemental material — Table S1; Fig. S1 and S2. [file spectrum.01176-24-s0001.docx]

### **Supporting Information**

**Dynamics of microbial induced oil degradation at the microscale**

Hong Zhang^1,2^^†^, Wenchao Zhang^3†,^, Yiwu Zong^1^ ,Dongyang Kong^1^, Luyan Ma^4#^ , Xiao-Lei Wu^5#^ , Kun Zhao^6,7#^

^1^Frontiers Science Center for Synthetic Biology and Key Laboratory of Systems Bioengineering (Ministry of Education), School of Chemical Engineering and Technology, Tianjin 300072, China

^2^Petrochemical Research Institute of Petrochina Co., Ltd.,Beijing 102206, China

^3^School of Chemistry and Life Science, Suzhou University of Science and Technology, Suzhou 215009, China

^4^State Key Laboratory of Microbial Resources Institute of Microbiology, Chinese Academy of Sciences, Beijing 10080, China

^5^College of Engineering, Peking University, Beijing 10080, China

^6^Institute of Fundamental and Frontier Sciences, University of Electronic Science and Technology of China, Chengdu, Sichuan 610054, China

^7^Sichuan Provincial People's Hospital, University of Electronic Science and Technology of China, Chengdu, Sichuan 610054, China

†These authors contributed equally to this work.

Table S1 Strains used in this study

| Strain | Genotype and/or relevant characteristics | Source or reference |
| --- | --- | --- |
| *P. aeruginosa* PAO1 | Wild type | Lab stock |
| *P. aeruginosa* O-2-2 | Wild type | Lab stock |
| *P. aeruginosa* IMP68 | Wild type | Ma *et al*，2020 ^[1]^ |
| *Dietzia* sp. DQ12-45-1b | Wild type | Lab stock |


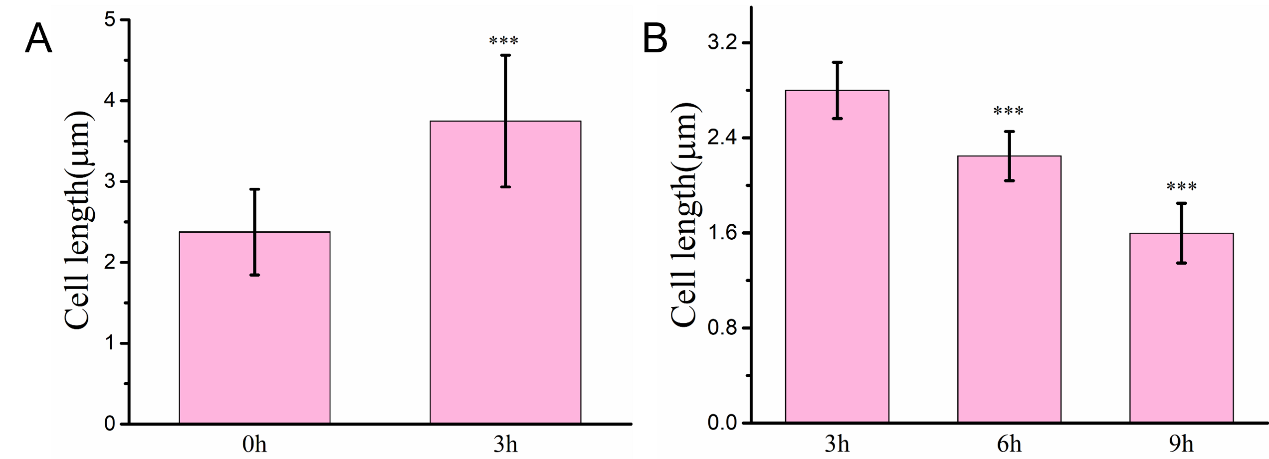


Fig S1 A) Cell length of *P. aeruginosa* O-2-2 measured at t = 0h and t = 3h before biofilm formation on hexadecane oil droplets in MSM medium supplemented with additional yeast extract (5 g/L). The analysis of statistical significance was performed using a two-sample Student’s *t*-test, *** p < 0.001; B) Cell length of O-2-2 that were attached on a glass surface in a flow-cell device supplemented with FAB medium and 0.6mM glutamate, measured at t = 3h, 6h, and 9h in the course of biofilm development. Statistical significances were measured using a one-way analysis of variance (ANOVA) set for multiple comparisons with a Dunnett's post-test. *** p < 0.001. The analysis of statistical significance was performed between 3h and other time points.


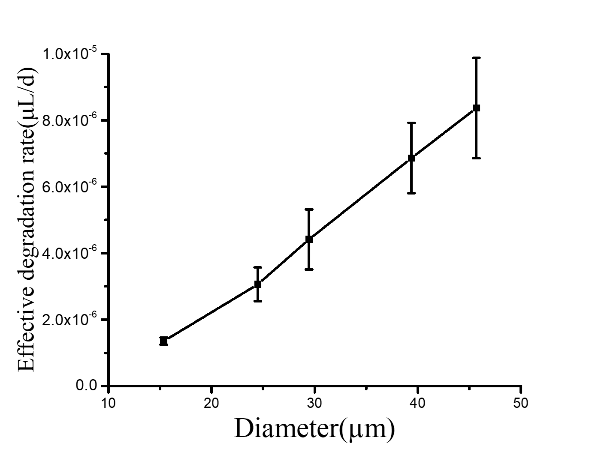


Fig S2 The size effect of oil droplets on the effective degradation rate of *P. aeruginosa* O-2-2

Supplementary references

[1] Xu A, Wang D, Ding Y, Zheng Y, Wang B, Wei Q, Wang S, Yang L, Ma LZ. 2022. Integrated comparative genomic analysis and phenotypic profiling of *Pseudomonas aeruginosa* isolates from crude oil. Front Microbiol 11: 519.
